# Gut to brain: essential micronutrient and trace element manganese transport, function and toxicity

**DOI:** 10.3389/fphys.2025.1651151

**Published:** 2025-12-16

**Authors:** Jiaqi Zou, Riya Yerramilli, Tolunay Beker Aydemir

**Affiliations:** Division of Nutritional Sciences, Cornell University, Ithaca, NY, United States

**Keywords:** ZIP14, SlC39A14, ZIP8, SLC39A8, SLC30A10, ZNT10, metal transport, Parkinsonism

## Abstract

This review provides a comprehensive analysis of manganese (Mn) metabolism and its regulatory roles across multiple biological levels. By examining Mn homeostasis mechanisms, including Mn absorption, excretion, distribution, and transport across the intestines, liver, and brain, this work highlights the integrative nature of Mn physiology. Additionally, it explores routes of Mn overexposure and the consequences of Mn dysregulation on various organ systems, with a focus on neurotoxicity, as well as the genetic and environmental factors that contribute to Mn homeostasis. This review synthesizes insights into metal transporters to advance our understanding of their roles in maintaining systemic and brain Mn homeostasis under healthy conditions and their contribution to Mn dysregulation in disease states, particularly neurological disorders. By focusing on Mn transport and regulation across multiple physiological systems and its impact on health and disease, we aim to bridge the gap between molecular-level processes and whole-body physiology.

## 1 Introduction

Mn is an essential trace element vital for diverse biological processes, including enzymatic catalysis, antioxidant defense, and neurotransmission. As a cofactor for enzymes such as Mn superoxide dismutase (MnSOD), arginase, and pyruvate carboxylase, Mn contributes to antioxidant defense, amino acid catabolism, and carbohydrate metabolism (Smith et al., 2010). Despite its importance, Mn levels in the body are tightly regulated, as both deficiency and excess can disrupt physiological functions and lead to disease. The gastrointestinal tract plays a key role in Mn uptake, mediated by metal transporters, and these transporters can also contribute to Mn excretion *via* the gastrointestinal tract and the hepatobiliary system (Aydemir et al., 2020; Mercadante et al., 2019; Taylor et al., 2019; Choi et al., 2024a; Chua and Morgan, 1997). The brain, particularly regions involved in motor function, is acutely sensitive to Mn overload, with dysregulation linked to Mn-induced Parkinsonism, a debilitating and progressive neurological disorder (Iqbal et al., 2012; Bjørk et al., 2017; Rutchik et al., 2012; Racette et al., 2001; Racette et al., 2012; Bowler et al., 2011; Jiang et al., 2006; Fitzgerald et al., 1999; Guilarte and Gonzales, 2015; Zhang et al., 2017; Vlasak et al., 2023; Tsuji et al., 2023).

Emerging research highlights the role of Mn beyond individual organ systems, revealing coordinated interactions between the liver, intestines, and brain. Disruptions in Mn balance impact not only local processes but also broader systemic networks, emphasizing its dual nature as both a necessary micronutrient and a potential neurotoxin. This review explores the physiological roles of Mn, its metabolism, key transporters, and associated genetic mutations, as well as an in-depth review of the routes and mechanisms of Mn toxicity in the brain. It also highlights current research gaps and future directions in Mn homeostasis. By addressing the integrative roles of Mn, the review aims to deepen our understanding of micronutrient regulation in both health and disease.

## 2 What is Mn, and what are its roles in the body?

Mn is the 12th most abundant element in the Earth’s crust. It is also an essential trace mineral found in the body. Mn finds its way into the body mainly through absorption in the gastrointestinal tract (Chen P. et al., 2018). The main sources of dietary Mn are drinking water and Mn-rich foods, such as whole grains, rice, nuts, and multivitamins (O’Neal and Zheng, 2015). In infants, breast milk and infant formula are the main sources of Mn (Stastny et al., 1984; Aschner and Aschner, 2005). Mn can also be absorbed in small amounts through the lungs, typically under high environmental exposure (Evans and Masullo, 2023; Karyakina et al., 2022).

Mn is an essential metal found in many metalloproteins, including arginase, glutamine synthetase, pyruvate carboxylase, and MnSOD (Smith et al., 2010). Mn is necessary for a wide range of cellular activities, as these enzymes are involved in the urea cycle, amino acid metabolism, carbohydrate metabolism, and antioxidant activity. Mn is a cofactor for MnSOD, the primary scavenger of reactive oxygen species (ROS) in the mitochondria (Holley et al., 2011). MnSOD is essential for life, as the knockout (KO) of MnSOD is lethal to neonatal mice (Li et al., 1995; Ikegami et al., 2002), while partial KO results in oxidative stress and DNA damage (Van Remmen et al., 2004; Liang and Patel, 2004; Ramachandran et al., 2011). In recent years, scientists have focused their research on the role of Mn in glycosylation, a common protein modification. Mn has been identified as essential for glycosylation on the Golgi apparatus *via* the Golgi calcium importer, TMEM165 (Powers et al., 2023; Liu et al., 2021). Potelle et al. (2016) demonstrated that Golgi protein TMEM165 is a potential Mn importer, as depletion of this transporter leads to reduced Mn levels and impaired glycosylation. Notably, these effects were reversed through Mn supplementation.

Mn also plays a crucial role in immunity beyond its function in metalloproteins. As part of nutritional immunity, the host restricts bacterial access to Mn by either sequestering Mn with calprotectin or by increasing zinc (Zn) levels, which compete with Mn for bacterial uptake, both of which can lead to Mn starvation in bacteria and inhibit bacterial growth (Morey et al., 2015). Additionally, Mn serves as an immune-stimulating agent. During infection, it acts as a ‘danger signal’, triggering apoptosis and the release of proinflammatory cytokines to help contain pathogen proliferation (Wang et al., 2020). Studies on Mn deficiency in humans and animals have demonstrated its essential role in reproduction, skeletal development, lipid metabolism, blood clotting, and blood glucose regulation (Powers et al., 2023; Finley and Davis, 1999).

## 3 Transmembrane transport of Mn and systemic regulation

In this review, we separate transmembrane transport at the cell-level uptake/efflux across polarized membranes (e.g., enterocytes, hepatocytes, brain endothelium), and systemic regulation at organ-level fluxes that determine how Mn moves among the intestine, liver, blood, and brain.

### 3.1 Transmembrane transport of Mn

#### 3.1.1 Intestinal metal transporters in Mn homeostasis

**FIGURE 1:**
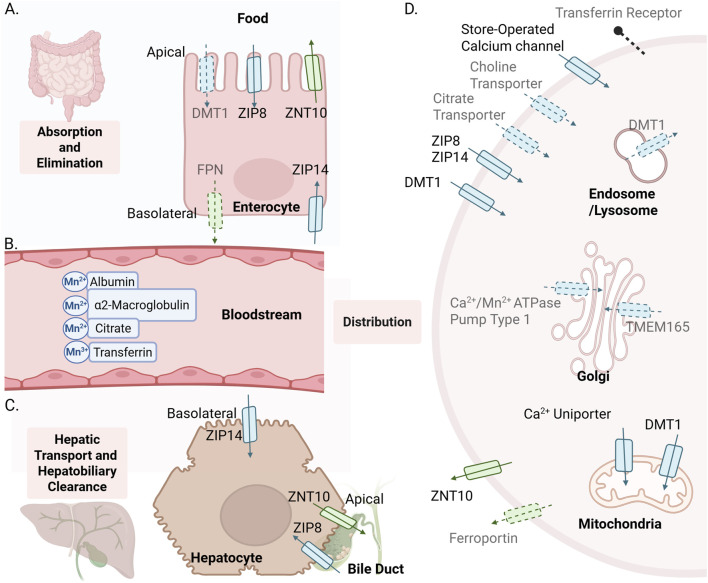
Systemic regulation of Mn. Confirmed Mn transporters supported by *in vivo* evidence are shown in solid color with defined directionality, whereas dashed arrows and shaded labels indicate proposed mechanisms based primarily on *in vitro* studies, often performed at supraphysiologic Mn concentrations. **(A)** The intestine regulates Mn through both absorption and excretion *via* transporters localized on the apical (lumen-facing) and basolateral (blood-facing) membranes of enterocytes. **(B)** In circulation, Mn exists mainly as Mn^2+^ bound to α_2_-macroglobulin, albumin, and citrate, with a minor fraction as transferrin-bound Mn^3+^. **(C)** In hepatocytes, ZIP14 on the basolateral membrane mediates Mn uptake from blood, ZNT10 on the apical membrane exports Mn into bile, and ZIP8 on the apical bile-canalicular membrane enables Mn reuptake from bile. **(D)** ZIP14, ZIP8, DMT1, and ZNT10 mediate the majority of Mn transport, supported by both *in vitro* and *in vivo* studies, while other pathways contribute to a lesser extent. Only a small proportion of Mn—Mn^3+^ bound to transferrin enters cells *via* transferrin receptor (Tfr)–mediated endocytosis. ZIP, Zrt-Irt-like Protein; ZnT, Zinc Transporter; DMT1, Divalent Metal Transporter 1; TMEM165, Transmembrane Protein 165.

Maintaining Mn within a narrow physiological range is important as both deficiency and excess can disrupt cellular function (Roth J. et al., 2013). Given that dietary intake is the primary source of Mn, the intestines serve as a major site of Mn regulation (Kippler and Oskarsson, 2024). The intestines regulate Mn through both absorption and excretion, which is controlled by specific transporters localized on the apical (lumen-facing) and basolateral (blood-facing) membranes of the intestinal epithelium (Figure 1A).

DMT1 is a divalent metal transporter primarily recognized for its role in iron (Fe) transport; however, emerging evidence suggests it may also mediate Mn uptake. It is located on the apical membrane of the duodenum and has been proposed to mediate dietary Mn absorption (Morgan and Oates, 2002; Mackenzie and Garrick, 2005). Studies on Belgrade rats with a DMT1 mutation demonstrated impaired intestinal Mn absorption; however, Fe deficiency may complicate these findings (Chua and Morgan, 1997). Moreover, a recent study in *Znt10* KO mice, a model of systemic Mn overload, shows that intestinal *Dmt1* deficiency reduced systemic Mn accumulation, indicating that DMT1 contributes to Mn overload in these mutant mice by promoting intestinal Mn absorption (Prajapati et al., 2025). Yet, *Dmt1* deficiency had little impact on Mn levels in wild-type mice under Mn-sufficient or Mn-rich diets (Prajapati et al., 2025). Consistent with this, a study reported that intestinal-specific deletion of *Dmt1* did not alter Mn absorption when Fe-adequate conditions (Shawki et al., 2015), highlighting a complex relationship between Fe status and Mn transport. The discrepancy in findings could stem from differences in experimental conditions: the former study used younger, developing animals fed on a lower-Fe diet (72 ppm), whereas the latter used mature adult mice fed a diet with nearly five-fold higher Fe content (350 ppm) (Shawki et al., 2015; Wang et al., 2018). Future studies should use consistent dietary Fe levels and developmental stages across models to clarify how DMT1 contributes to Mn absorption.

While DMT1 contributes to Mn transport in the intestines, additional transporters are likely involved. ZIP8 is a member of the ZIP family, known to transport divalent metals such as Zn, Fe, and cadmium (Cd) (He et al., 2006; Knutson, 2019). Its role in Mn homeostasis was first discovered through mutations in human homolog *SLC39A8*, which resulted in severe systemic Mn deficiency (Riley et al., 2017; Boycott et al., 2015; Park et al., 2015; Winslow et al., 2020). Similar to DMT1, ZIP8 is localized at the apical membrane of the intestinal epithelium (Melia et al., 2019; Choi et al., 2024b). However, relatively few studies have explored its contribution to intestinal Mn absorption. Only one study has reported that intestinal-specific *Zip8* KO mice exhibited impaired intestinal Mn uptake and systemic Mn deficiency, supporting its role in transcellular Mn uptake from the lumen into enterocytes (Choi et al., 2024b).

While more is known about the transporters that handle Mn uptake into enterocytes, less is understood about which transporter exports Mn from enterocytes into the bloodstream on the basolateral membrane. Ferroportin (FPN), a divalent metal exporter, has been suggested as a possible candidate, as shown by its basolateral localization on the enterocytes (Han and Kim, 2007) and impaired intestinal Mn absorption in *Fpn* KO mice (Seo and Wessling-Resnick, 2015). However, other studies indicate human FPN to be an inefficient transporter of Mn, with both low efflux rate and low affinity compared to other metals (Mitchell et al., 2013; Li et al., 2020). Therefore, further research is needed to identify and characterize the primary basolateral exporter on enterocytes.

Comparatively, transporters involved in Mn excretion from the intestines are better characterized. ZIP14, structurally similar to ZIP8, transports several divalent metals, including Mn, from the extracellular or organelle spaces into the cell cytosol (Tuschl et al., 2016; Taylor et al., 2005; McCabe and Zhao, 2024; Steimle et al., 2019; Hendrickx et al., 2018). ZIP14 is the key transporter for Mn excretion and is located on the basolateral membrane of enterocytes. Our previous study using intestine-specific *Zip14* KO (IKO) mice revealed that intestinal ZIP14 mediates basolateral uptake of Mn from the bloodstream into enterocytes, contributing to Mn excretion into the intestinal lumen (Aydemir et al., 2020). When radioactive ^54^Mn was administered subcutaneously, intestinal radioactivity was significantly lower in IKO mice compared to controls, indicating impaired basolateral-to-apical Mn transport. In contrast, oral delivery of ^54^Mn resulted in no difference between IKO and controls, suggesting that ZIP14 is not essential for apical Mn absorption from the lumen (Aydemiret al., 2020). These findings were supported by polarized Caco2 cells, where *Zip14* KO cells showed almost no uptake of ^54^Mn from the basolateral side but retained normal uptake from the apical side (Scheiber et al., 2019).

Another key player in Mn excretion is ZNT10. Although structurally similar to other ZNT family members that transport Zn, ZNT10 also functions as a Mn exporter (Nishito et al., 2016; Zogzas and Mukhopadhyay, 2018). This transporter is localized on the apical membrane of enterocytes, as shown by immunofluorescence staining on Caco2 cells in a transwell system, and mediates Mn efflux into the lumen (Taylor et al., 2019). Intestinal-specific *Znt10* KO mice have significantly greater Mn levels in their duodenum tissue, suggesting impaired Mn export out of enterocytes (Mercadante et al., 2019).

Together, these transporters coordinate intestinal Mn absorption and excretion, making the intestines a crucial site for systemic Mn regulation.

#### 3.1.2 Hepatic metal transporters in Mn homeostasis

Similar to the intestines, ZIP14, ZIP8, and ZNT10 also participate in Mn homeostasis in liver hepatocytes (Figure 1C). ZIP14 is localized on the basolateral membrane of the hepatocyte, facing the portal vein (Thompson and Wessling-Resnick, 2019) and facilitates Mn uptake. Transport studies in polarized HepaRG cells show that ZIP14-mediated Mn transport is downregulated in the presence of high extracellular Mn (Thompson and Wessling-Resnick, 2019). Western blot analysis confirmed that ZIP14 protein levels decreased with increasing Mn exposure. This downregulation could be a protective mechanism to limit Mn uptake and maintain cell viability. Additionally, the trend was not observed for the other transporter proteins: ZNT10 and ZIP8. The lack of response in other transporters highlights ZIP14’s unique sensitivity and role in Mn homeostasis. In addition, recent studies using liver-specific *Zip14* KO mice displayed an almost complete loss of hepatic Mn, demonstrating that ZIP14 is the primary transporter responsible for Mn uptake into the liver (Xin et al., 2017; Fung and Zhao, 2022).

ZNT10 is located on the apical membrane, facing the bile canaliculi, of the hepatocyte and is known to excrete Mn out of the cell and into the bile. In 6-week-old *Znt10* KO mice, liver Mn levels were significantly elevated (ranging from twenty to sixty-fold increase) compared to WT (Liu C. et al., 2017; Hutchens et al., 2023). Furthermore, in the Hep3B cell line, the *Znt10* mutant cells were significantly more susceptible to Mn toxicity compared to the control. This revealed that the ZNT10 transporter plays a key role in clearing Mn from the cell to prevent toxicity from high levels of Mn. These findings were further confirmed by transfecting the mutant cells with a plasmid expressing the wild-type (WT) *Znt10* gene, which showed partial mediation of the Mn toxicity in the mutant cells. Both the human *SLC30A10* mutant Hep3B cell line and primary mouse hepatocytes with Z*nt10* KO showed a significant impairment in Mn export when compared to WT (Prajapati et al., 2021).

ZIP8 is localized on the apical membrane of mouse hepatocytes, specifically in the bile canaliculi, where it plays a key role in Mn reuptake from the bile (Lin et al., 2017; Sunuwar et al., 2020). Hepatic-specific *Zip8* KO mice showed significantly lower hepatic Mn levels (Powers et al., 2023), as did *Zip8* loss-of-function mutations (Sunuwar et al., 2020), while bile Mn levels were increased by two-fold (Sunuwar et al., 2020).

### 3.2 Systemic regulation of Mn

Mn exists mainly as two oxidation states in the body: Mn^2+^, the stable and more dominant form, and Mn^3+^, a powerful oxidant present at much lower levels (Roth J. et al., 2013; Kippler and Oskarsson, 2024). Mn enters the body primarily through two routes: dietary intake and inhalation. Dietary Mn is mainly present in foods such as whole grains, fruits, and nuts, predominantly as Mn^2+^ bound to organic acids, proteins, and cell wall components, rather than as free ions (Schmidt and Husted, 2019; Kuang et al., 2022). Mn salts in the form of Mn^2+^ can also be added to food and dietary supplements (EFSA Panel on Dietetic Products and Nutrition and Allergies NDA, 2013). During digestion, it has been proposed that gastric acidity helps release Mn^2+^, making it bioavailable for absorption by intestinal transporters (Silva et al., 2020). In contrast, excess environmental Mn, typically present as inorganic oxides in fumes or dust, can be absorbed *via* the lungs or nasal route (Evans and Masullo, 2023; Karyakina et al., 2022).

Once in circulation, Mn exists mainly as Mn^2+^ bound to α2-macroglobulin and albumin, with small fractions bound to citrate or as transferrin-bound Mn^3+^ (Roth J. et al., 2013; Kippler and Oskarsson, 2024) (Figure 1B). Blood Mn levels typically range between 2.8 and 15.4 μ g/L (Liu et al., 2021; Rabin et al., 1993; Yokel, 2002; Williams et al., 2012). Mn is distributed across multiple tissues, including the liver (1.32 mg/kg), pancreas (1.17 mg/kg), bones (1 mg/kg), and kidney (0.98 mg/kg) (O’Neal and Zheng, 2015; Rahil-Khazen et al., 2002). The brain is also a major site of Mn deposition, with regional concentrations ranging from 0.16 to 0.46 mg/kg, the highest levels found in the globus pallidus and putamen, regions primarily responsible for motor control (Krebs et al., 2014).

The liver and intestines work together to ensure tight regulation of systemic Mn levels. Under Mn excess, hepatic ZIP14 takes up Mn from the blood into hepatocytes (Nam et al., 2013; Guthrie et al., 2014; Thompson et al., 2018; Thompson and Wessling-Resnick, 2019) and excretes it into the bile *via* ZNT10. Mn is then transported to the intestines *via* the hepatobiliary system and excreted into feces (Mercadante et al., 2019; Taylor et al., 2019; Liu C. et al., 2017). The intestines can also excrete Mn directly into the intestinal lumen by the same duo, ZIP14 and ZNT10 (Aydemir et al., 2020; Mercadante et al., 2019; Taylor et al., 2019; Hutchens et al., 2023). Recent studies demonstrate that intestinal and hepatic transporters play compensatory roles in Mn excretion. Hepatic *Zip14* deletion alone did not affect systemic Mn levels (Xin et al., 2017), but double KO of both hepatic and intestinal *Zip14* did result in significant systemic Mn overload (Fung and Zhao, 2022; Hutchens et al., 2023). Similarly, intestine-specific *Znt10* KO revealed moderate systemic Mn accumulation, whereas intestine and liver double *Znt10* KO resulted in severe Mn overload compared to either KO alone (Mercadante et al., 2019), suggesting that both intestine and liver ZNT10 play an essential role in Mn excretion.

While much attention has been given to ZIP14 and ZNT10, hepatic ZIP8 also plays a critical role in systemic Mn regulation. Mutant ZIP8 mice exhibited systemic Mn deficiency, with significant reductions in Mn levels in whole blood, liver, and kidney (Sunuwar et al., 2020). Furthermore, a study revealed that liver-specific *Zip8* KO mice exhibited significant decreases in systemic Mn levels under both Mn-rich and Mn-depleted conditions, indicating that hepatic ZIP8 mediates Mn reabsorption from bile to help maintain systemic homeostasis (Powers et al., 2023). Notably, a recent study showed that neonatal mouse pups caused significant downregulation of hepatic ZIP8 under a high-Mn diet, increasing hepatobiliary Mn excretion. This suggests a protective mechanism against Mn overload in neonates, a vulnerable developmental stage (Yu and Zhao, 2023). This regulatory response was absent in adult mice (Yu and Zhao, 2023), highlighting age-dependent variations in systemic Mn regulation that should be considered in future studies.

### 3.3 Genetic mutations associated with Mn dyshomeostasis

Mutations in transporters of Mn can disrupt Mn homeostasis, leading to either deficiency or overload. Human genetic studies have been instrumental in understanding the function of these transporters.

Homozygous missense mutations in *SLC39A8*, which encodes ZIP8, result in Mn deficiency in humans (Riley et al., 2017; Boycott et al., 2015; Park et al., 2015; Winslow et al., 2020). This can be attributed to the absence of ZIP8 in enterocytes and bile canaliculi, reducing Mn absorption from the intestines and reabsorption from the bile, thereby indirectly leading to more Mn excretion. Typically, onset occurs at birth or early infancy, with blood Mn levels by 5–10 times lower than normal (Riley et al., 2017; Boycott et al., 2015; Park et al., 2015; Winslow et al., 2020). Symptoms of Mn deficiency include short stature, cognitive impairments, developmental delays, cerebellar atrophy, and hypotonia, often appearing at birth or in early childhood (Riley et al., 2017; Boycott et al., 2015; Park et al., 2015; Winslow et al., 2020). A recent study characterized 21 human *SLC39A8* variants and further demonstrated that different missense mutations can variably impair Mn uptake, linking specific genetic variation to functional deficits (Wang et al., 2025).

In contrast, homozygous missense and deletion mutations in *SLC3914*, the gene encoding ZIP14, cause systemic Mn overload and hypermanganesemia (Tuschl et al., 2016; Juneja et al., 2018; Marti-Sanchez et al., 2018; Rodan et al., 2018; Zhang et al., 2023; Zeglam et al., 2019). Blood Mn levels are typically more than ten times greater than the normal range. The absence of ZIP14, particularly in hepatocytes and enterocytes, impairs Mn elimination, preventing excretion *via* the hepatobiliary and intestinal routes and leading to systemic Mn accumulation, including the brain. Similar to *SLC39A8* mutations, symptoms manifest within the first few years of life (Winslow et al., 2020). Individuals with this condition exhibit Parkinsonian features, including dystonia, tremors, bradykinesia, and abnormal gait. Notably, Fe and Zn in these patients remain normal, suggesting that *SLC39A14* deletion specifically affects Mn (Tuschl et al., 2016; Juneja et al., 2018; Marti-Sanchez et al., 2018; Rodan et al., 2018; Zhang et al., 2023; Zeglam et al., 2019).

Mutations in *SLC30A10*, which encodes ZNT10, also cause hypermanganesemia, with elevated Mn levels in the blood and brain. The mutations include deletion, missense, heterozygous, and exon deletions, resulting in blood Mn concentrations 5–20 times above the normal range (Winslow et al., 2020; Zaki et al., 2018; Quadri et al., 2012; Tuschl et al., 2012; Quadri et al., 2015; Tavasoli et al., 2019; Gulab et al., 2018; Yapici et al., 2019). Since ZNT10 is responsible for excreting Mn from the liver and intestines, its absence leads to Mn accumulation in both the liver and the bloodstream. Unlike *SLC39A8* and *SLC39A14* mutations, symptoms of *SLC30A10* mutations can emerge in childhood or adulthood (Winslow et al., 2020; Zaki et al., 2018; Quadri et al., 2012; Tuschl et al., 2012; Quadri et al., 2015; Tavasoli et al., 2019; Gulab et al., 2018; Yapici et al., 2019). Affected individuals experience dystonia, gait abnormalities, and speech disturbances, along with distinct features such as polycythemia and liver disease (Zaki et al., 2018; Quadri et al., 2012; Tuschl et al., 2012; Quadri et al., 2015; Tavasoli et al., 2019; Gulab et al., 2018; Yapici et al., 2019). Polycythemia may result from elevated Mn levels stimulating erythropoietin production, which promotes red blood cell synthesis (Quadri et al., 2015). Chelation therapy is a potential treatment option as it binds to excess metals, facilitating their removal from the body. It has been shown to improve clinical symptoms and reduce brain Mn accumulation (Stamelou et al., 2012).

### 3.4 Mn distribution within cells

Cellular Mn uptake can be mediated through multiple pathways (Figure 1D). The majority of Mn, primarily as Mn^2+^, carried by globulin and albumin in the serum, enters the cell *via* transmembrane transporters such as ZIP8, ZIP14, and DMT1. These transporters are expressed on the plasma membrane and import Mn, in addition to other divalent metals (Nishito et al., 2016; Tuschl et al., 2012; Quadri et al., 2015; Tavasoli et al., 2019; Gulab et al., 2018; Yapici et al., 2019; Stamelou et al., 2012; Fujishiro et al., 2012; Wang et al., 2012; Jeong and Eide, 2013). Only a small fraction of Mn, in the form of Mn^3+^ bound to transferrin, enters the cell through transferrin receptor-mediated endocytosis (Gunter et al., 2013). In the brain, other transporters have been observed to take up Mn into cells. Calcium channels have been shown to mediate Mn uptake both *in vitro* and *in vivo*, as Mn uptake was enhanced by calcium channel activators and inhibited by blockers (Crossgrove et al., 2005). More recently, mice lacking the L-type calcium channel had reduced Mn entry into neurons (Bedenk et al., 2018). Choline transporters may be another pathway into the brain, as demonstrated by Mn^2+^-dependent inhibition of choline uptake in rats; however, no recent studies have explored this mechanism further (Lockman et al., 2001). Additionally, citrate-bound Mn (about 2% of plasma Mn) may enter the brain *via* a shared citrate transporter, as its influx exceeded diffusion and the transporter was competitively inhibited by Mn citrate (Crossgrove et al., 2003). While these pathways have been observed under experimental conditions, their contribution to physiological Mn uptake is likely smaller than that of ZIP14, ZIP8, and DMT1 and should be quantified in future studies.

Intracellularly, both DMT1 (Wolff et al., 2018) and mitochondrial calcium uniporter (Gavin et al., 1999; Kamer et al., 2018) can participate in Mn^2+^ uptake into the mitochondria *in vitro* (Figure 1D). In the Golgi apparatus, TMEM165 (Potelle et al., 2016) and SPCA1 (Leitch et al., 2011; Mukhopadhyay and Linstedt, 2011) are suggested to transport Mn^2+^ *in vitro*. For transferrin-bound Mn^3+^, Mn^3+^ is released in endosomes and lysosomes, reduced to Mn^2+^, and exported to the cytosol *via* DMT1 *in vitro* (Au et al., 2008) (Figure 1D). Conversely, Mn^2+^ export is primarily mediated by ZNT10 (Leyva-Illades et al., 2014; Nishito et al., 2016). Ferroportin is also proposed to export Mn^2+^ (Seo and Wessling-Resnick, 2015; Choi et al., 2019; Li et al., 2013; Madejczyk and Ballatori, 2012). Mutations in ferroportin resulted in Mn accumulation and cytotoxicity *in vitro* (Choi et al., 2019). However, a recent study reported that neither human nor mouse ferroportin exported Mn from oocytes expressing the ferroportin gene, and Mn efflux remains unchanged regardless of ferroportin expression, challenging its role in Mn transport (Azucenas et al., 2023).

Overall, ZIP14, ZIP8, DMT1, and ZNT10 appear to mediate the majority of Mn transport, supported by extensive *in vitro* and *in vivo* evidence, with other pathways contributing to a lesser extent.

## 4 Mn metabolism in the brain

### 4.1 Role of Mn in the brain

Under normal physiological conditions, Mn serves as a vital cofactor for Mn-dependent enzymes in the brain. The most important among these enzymes are glutamine synthetase, MnSOD, and arginase. Glutamine synthetase, found in astrocytes, converts glutamate into glutamine. It is essential for the glutamate-glutamine cycle, a process where astrocytes rapidly remove glutamate released by the neurons and replenish neurons with glutamine (Bak et al., 2006). This cycle prevents glutamate excitotoxicity caused by excess glutamate, which can lead to neuronal damage and death. However, as discussed below, excess Mn exposure can also negatively affect this cycle. MnSOD, located in the mitochondria, is an antioxidant enzyme that converts superoxide anion into hydrogen peroxide, thereby lowering oxidative stress. In the brain, MnSOD can protect neurons from hypoxia and brain injury (Keller et al., 1998; Xiong et al., 2005; Shan et al., 2007). Nevertheless, similar to glutamine synthetase, elevated levels of Mn can reduce MnSOD expression. Lastly, arginase is an enzyme in the urea cycle, responsible for converting arginine to ornithine and urea. It is found in neurons and glial cells in most brain areas. Particularly relevant in the brain, arginase serves as a substrate for nitric oxide synthase, which produces nitric oxide–an important cellular messenger (Wiesinger, 2001). In this role, arginase can protect against brain injury (Madan et al., 2018; Fouda et al., 2020).

### 4.2 Mn routes into the brain

**FIGURE 2:**
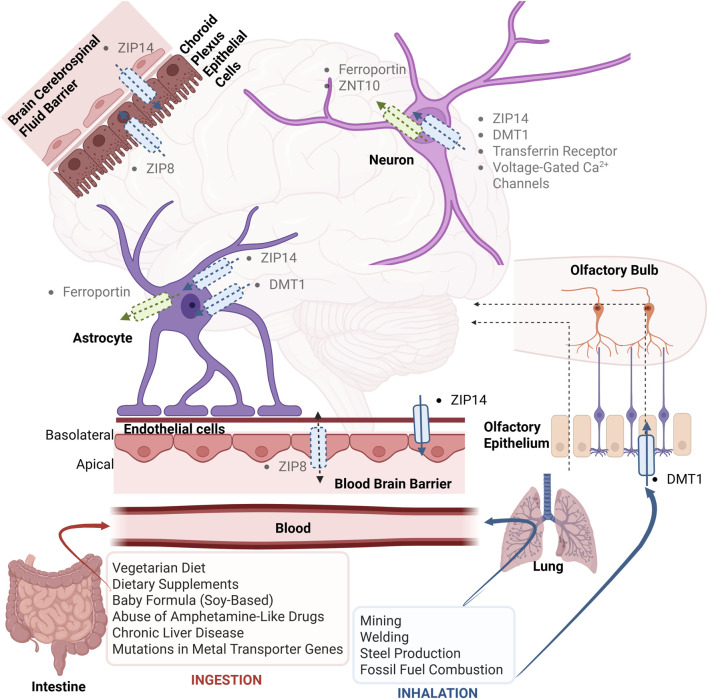
Mn transport dynamics in the brain. Confirmed Mn transporters supported by *in vivo* evidence are shown in solid color with defined directionality. Dashed arrows and shaded labels indicate proposed mechanisms based primarily on *in vitro* studies, often conducted at supraphysiologic Mn concentrations. ZIP14, localized basolaterally, mediates Mn efflux from the brain to the blood, whereas ZIP8, present on both apical and basolateral membranes, may facilitate bidirectional Mn transport, although its *in vivo* polarity remains unresolved. DMT1, TfR, ferroportin, ZNT10, and voltage-gated Ca^2+^ channels are shown as additional potential Mn transport routes, though their physiological relevance is less established. ZIP, Zrt-Irt-like Protein; ZnT, Zinc Transporter; DMT1, Divalent Metal Transporter 1.

Mn can enter the brain *via* three routes: the blood-brain barrier (BBB), the blood-cerebrospinal fluid barrier, and the olfactory nerve terminals (Figure 2). Once in the brain, Mn accumulates primarily in the basal ganglia, especially in the globus pallidus (Guilarte et al., 2006a; Newland et al., 1989; Dorman et al., 2006). The basal ganglia are a collection of subcortical nuclei that play a role in motor control. Pathological changes in the basal ganglia are linked to various movement disorders, such as Parkinson’s Disease, hyperkinetic movement disorders, dystonia, and Tourette’s Syndrome (Fazl and Fleisher, 2018).

### 4.3 Mn transport dynamics at the BBB

The brain tightly regulates substances that enter and exit. The BBB stands between systemic blood circulation and the brain parenchyma. The BBB consists of endothelial cells held by tight junctions and adherens junctions, a basement membrane that serves as a scaffold, pericytes that surround the vessels, and astrocytic end feet encircling them both (Maiuolo et al., 2018). Tight junctions include occludin, claudin, and junctional adhesion molecules (Maiuolo et al., 2018). This tight network collaborates to restrict large molecules from entering by the paracellular route. Only small, highly lipophilic molecules can passively diffuse across the barrier (Maiuolo et al., 2018). Therefore, metal ions can only enter and exit the brain *via* transporters on the endothelial cells.

#### 4.3.1 BBB endothelial cells have polarity

The apical side of the BBB faces the blood, whereas the basolateral side comes into contact with the basement membrane, astrocytes, and pericytes. Similar to intestinal enterocytes, brain endothelial cells exhibit apicobasal polarity, meaning that the apical and basolateral plasma membranes possess different protein and lipid compositions (Worzfeld and Schwaninger, 2016). This polarity is critical for controlling the movement of hydrophilic substances such as glucose, amino acids, metals, and drugs (Maiuolo et al., 2018; Tang et al., 2012). Polarity is initially established during angiogenesis as an embryo (Worzfeld and Schwaninger, 2016; Potente et al., 2011). This characteristic is a vital aspect in understanding Mn transport across the BBB since Mn transporter expression could be different on the apical *versus* the basolateral side.

Studies have suggested that excess Mn can compromise BBB integrity by increasing leakiness and reducing tight junction proteins (Oliveira-Paula et al., 2024; Sharma et al., 2021), with recent work pointing to disruption of β-catenin and Rho/ROCK signaling pathways (Wu et al., 2024; Ma et al., 2024). Interestingly, the impact of Mn overexposure on the integrity of the BBB appears to vary depending on exposure to the apical or basolateral side. Incubation of ≥ 200 µM MnCl_2_ for 72 h on the apical side led to reduced transepithelial/transendothelial electrical resistance (TEER) values and increased [14C] sucrose permeability (Bornhorst et al., 2012). In contrast, exposure of 500 µM MnCl_2_ to the basolateral side did not impair TEER values, nor result in [14C] sucrose permeability (Bornhorst et al., 2012). This suggests that the apical side is more susceptible to Mn toxicity, possibly due to the apicobasal distribution of Mn transporters. Consequently, manipulating Mn transporters has the potential to regulate Mn levels in the brain.

#### 4.3.2 Mn transporters on the BBB

ZIP8 and ZIP14 are expressed on brain endothelial cells (Steimle et al., 2019). Localization of ZIP8 and ZIP14 on the apical or basolateral membrane of BBB endothelial cells is important to know because these cells are polarized. Their localization provides insight into function: apical expression would support a role in Mn uptake into the brain, whereas basolateral expression would suggest a role in Mn efflux from the brain. In the Steimle study, the authors demonstrated the impact of ZIP8 and ZIP14 knockdown on Mn transport across the BBB by loading ^54^Mn into either the apical or basolateral side of transwells (Steimle et al., 2019). They found that knockdown of ZIP8 reduced transport in both directions, while knockdown of ZIP14 specifically affected the basolateral-to-apical transport. Double knockdown of ZIP8 and ZIP14 resulted in a more pronounced reduction in both directions. Cell surface biotinylation further showed ZIP8 and ZIP14 on both membranes of human brain microvascular endothelial cells (hBMVECs), with ZIP8 more abundant apically and ZIP14 basolaterally, though not statistically significant (Steimle et al., 2019). Collectively, these findings suggest, *in vitro*, *that* ZIP8 may contribute to both uptake and efflux of Mn, while ZIP14 may play a greater role in Mn efflux due to its basolateral enrichment and specific impact on basolateral-to-apical transport. ZIP8 and ZIP14 have been found on other cell types to display cell polarity (Steimle et al., 2019) (Figure 2). In intestinal enterocytes, which are also polarized, ZIP14 is expressed on the basolateral side (Aydemir et al., 2020) while in kidney cells, also polarized, ZIP14 and ZIP8 are expressed on the apical side (Girijashanker et al., 2008).

Our recent study comprehensively demonstrated ZIP14 expression in mouse brain sections, isolated brain microvessels, primary brain endothelial cells, and tubules formed by these cells (Zou et al., 2025). Notably, expansion microscopy of mouse brain sections showed that following Mn supplementation, ZIP14 expression shifted to greater expression on the basolateral side, supporting its role in basolateral-to-apical transport and Mn efflux from the brain (Maiuolo et al., 2018). Consistent with this, endothelial-specific *Zip14* KO mice exhibited impaired brain Mn efflux after nasal Mn supplementation and accumulated more Mn in the brain after Mn overexposure (Maiuolo et al., 2018). Additionally, in the transwell system, endothelial cells from KO animals showed reduced radioactive Mn uptake from the basolateral side, whereas human brain endothelial cells overexpressing ZIP14 had greater Mn transport from the basolateral-to-apical direction (Maiuolo et al., 2018). Together, these results highlight endothelial ZIP14 as a key mediator of Mn efflux, localized to the basolateral membrane of the BBB. Since *Zip14* KO did not completely inhibit efflux, other transporters, such as ZIP8, may also contribute, though *in vivo* studies are needed to confirm this.

Although the transferrin receptor (Tfr) is expressed in brain endothelial cells, studies suggest that Tfr is not required for Mn uptake across the BBB. In mice with hypotransferrinemia (transferrin levels <1%), the administration of ^54^Mn, either subcutaneously (Dickinson et al., 1996) or intravenously (Malecki et al., 1999), did not result in a significant difference in brain ^54^Mn accumulation when compared to control mice. These results suggest that the transferrin-Tfr system is not required for Mn uptake into the brain. A recent study investigated the apicobasal localization of Tfr in brain endothelial cells and discovered that Tfr primarily transports in the brain-to-blood direction (efflux) (Nielsen et al., 2023). However, as of yet, no research has examined the possible role of Tfr in the uptake of Mn in the blood-to-brain direction.

Similarly, DMT1 is not essential for Mn uptake across the BBB. *In situ* brain perfusion with ^54^Mn showed no difference in ^54^Mn uptake between Belgrade mice lacking DMT1 and control mice (Crossgrove and Yokel, 2004). In addition, one study found that ^54^Mn accumulation only happens at pH 7.8, whereas DMT1 functions at pH 5.5, making it unlikely that DMT1 contributes to Mn uptake (Steimle et al., 2019).

### 4.4 Other routes of Mn transport

#### 4.4.1 Blood-cerebrospinal fluid barrier

Another possible route for Mn entrance to the brain is through the blood-cerebrospinal fluid barrier (BCB). The BCB is the barrier between systemic blood and the cerebrospinal fluid (CSF) in the four ventricles of the brain. The BCB is comprised of choroid plexus capillaries, a basement membrane, and choroid plexus epithelial cells (Solár et al., 2020). Unlike the BBB, the BCB capillaries are fenestrated and permeable. Surrounding the capillaries is the basement membrane, which supports a layer of choroid plexus epithelial (CPE) cells that face the CSF. These CPE cells are responsible for producing and secreting the CSF. In contrast to BBB, where endothelial cells provide the tight barrier between blood and brain, CPE cells assume that role within the BCB (Lun et al., 2015). CPE cells are connected by tight junctions, which are responsible for maintaining barrier integrity. The tight junctions include ZO1, occludin, and claudins, which are also present in the BBB (Solár et al., 2020). CPE cells also have transporters that control the movement of materials from the blood to the CSF. The rest of the ventricle is lined by ependymal cells that are permeable to macromolecules (Johanson et al., 2011). Therefore, once substances enter the CSF, they can easily diffuse across the ependymal layer and into the brain. Thus, CPE cells serve as the primary line of defense between the choroid plexus capillaries and the brain.

It has been shown that Mn overexposure can negatively affect BCB tightness and function (Bornhorst et al., 2012). A study that examined Mn transport across the BCB discovered that when given equal amounts of Mn in a transwell system, Mn transport seems to be greater from the blood-to-brain direction than in the other direction (Bornhorst et al., 2012). This could be explained by the apicobasal polarity found in these CPE cells, similar to BBB endothelial cells (Gründler et al., 2013; Bernd et al., 2015; Rose et al., 2018; Häuser et al., 2018; Hochstetler et al., 2022; Vong et al., 2021). Among the polarized proteins are metal transporters that regulate metal movement across the BCB. One study found that ZIP14 and ZIP8 both participated in Mn transport across a choroid plexus cell line (Morgan et al., 2020). Knockdown of each transporter reduced ^54^Mn uptake into the cells. Furthermore, they confirmed that ZIP14 expression was enriched on the basolateral side and ZIP8 on the apical side (Figure 2). As for DMT1, although it is found to be present in the BCB (Wang et al., 2006), it does not seem to be a major transporter of Mn (Bornhorst et al., 2012). However, these studies were done *in vitro* and should be validated *in vivo*.

#### 4.4.2 Nasal route

Although the nasal route is not the main route for Mn entry into the brain, it becomes highly relevant under conditions of occupational or environmental inhalation overexposure. Mn in the air can be inhaled and enter the brain directly *via* olfactory pathways, by-passing the BBB. Mn predominantly enters the CNS through the faster extracellular route, where it is carried paracellularly *via* bulk flow to the CSF and subsequently diffuses into the brain (Crowe et al., 2018). Comparatively, less Mn is transported transcellularly through olfactory sensory neurons *via* metal transporters (Crowe et al., 2018). Therefore, few studies have investigated the metal transporters involved in Mn uptake in the nasal epithelium. Only one study found DMT1 expressed on the olfactory epithelium and whole-body deletion of DMT1 significantly reduced intranasal ^54^Mn uptake (Thompson et al., 2007) (Figure 2).

Since inhalation of toxic fumes is a major route of Mn overexposure in humans, intranasal delivery of Mn is widely used as an *in vivo* inhalation exposure model in research and has been shown to alter neurotransmitter levels, induce neuroinflammation, and impair behavior in mice (Moberly et al., 2012; Kaur and Prakash, 2020; Ivleva et al., 2022; Fa et al., 2010; Johnson et al., 2018; Pajarillo et al., 2020a; Ye and Kim, 2015; Dorman et al., 2004; Brenneman et al., 2000; Tjälve et al., 1996; Gianutsos et al., 1997).

### 4.5 Mn transporters in neurons and glia

Identifying transporters of Mn in neurons and glia is important, as these cells regulate brain Mn homeostasis and are primary targets of Mn neurotoxicity (Pajarillo et al., 2021). In neurons, evidence for Mn transporters is limited and often based on non-physiological conditions. Although DMT1 is expressed in neurons, its main role is Fe uptake (Pelizzoni et al., 2012; Skjørringe et al., 2015; Tai et al., 2016). Only one study suggested Mn transport *via* DMT1, but it used DMT1 expression and supraphysiological Mn exposure *in vitro* (Tai et al., 2016). Mn uptake through Tfr has been demonstrated through fluorescence-labeled Mn^3+^-bound transferrin complexes (Gunter et al., 2013); however, since very little circulating Mn (around 1%) is bound to transferrin (Kippler and Oskarsson, 2024), this route is unlikely to contribute significantly *in vivo*. Mn uptake *via* voltage-gated calcium channels in dopaminergic neurons has also been reported (Dučić et al., 2013), but again at high Mn concentrations. While these studies suggest possible routes of Mn uptake in neurons, their physiological relevance remains uncertain. ZIP14 and ZNT10 are expressed in human SH-SY5Y neuroblastoma cells. Under inflammatory conditions, ZIP14 expression is upregulated while ZNT10 is downregulated, accompanied by a three-fold increase in total Mn concentrations, suggesting that these two transporters play opposing roles in regulating Mn transport in neurons (Fujishiro et al., 2014) (Figure 2).

In astrocytes, ZIP14 expression has been demonstrated *in vivo* and *in vitro*, though only in the context of Fe uptake (Routhe et al., 2020) (Figure 2). Direct evidence for DMT1-mediated Mn transport is lacking, with only indirect evidence from an early study (Erikson and Aschner, 2006). Ferroportin is expressed in both neurons and astrocytes (Wu et al., 2004). While it is upregulated by Mn exposure (Yin et al., 2010), these studies used supraphysiological doses, and ferroportin likely functions primarily in Fe transport (Schulz et al., 2012; Aguirre et al., 2005; Ji et al., 2018). Transporters in microglial cells are largely unexamined. Although excess Mn can activate microglia and promote neuroinflammation (Tjalkens et al., 2017), astrocytes may be the more dominant mediator of Mn neurotoxicity in the brain. Much remains to be explored regarding the specific transporters in neurons and glia, which are critical for understanding how Mn overload contributes to neurotoxicity.

## 5 Mn toxicity

### 5.1 Mn overexposure

While Mn deficiency is rare, as it is found in various foods, Mn toxicity in humans is becoming increasingly prevalent. This rise in toxicity cases can be attributed to the high Mn levels in contaminated water, occupational inhalation of fumes, and injection of intravenous drugs (O’Neal and Zheng, 2015) (Figure 2). The overexposure of Mn in human cases has resulted in cognitive and motor deficits with symptoms similar to Parkinson’s Disease (Iqbal et al., 2012; Bjørk et al., 2017; Rutchik et al., 2012; Racette et al., 2001; Racette et al., 2012; Bowler et al., 2011; Jiang et al., 2006; Fitzgerald et al., 1999; Guilarte and Gonzales, 2015; Zhang et al., 2017; Vlasak et al., 2023; Tsuji et al., 2023).

#### 5.1.1 Oral Mn

Oral Mn exposure and its neurodevelopmental consequences have been well documented in children. Children exposed to elevated Mn levels in drinking water frequently experience developmental difficulties. Cross-sectional studies have linked high Mn concentrations in drinking water to attention deficits, increased aggression, and lower performance on math tests in children (Khan et al., 2011; Khan et al., 2012; Bouchard et al., 2011). Furthermore, a prospective cohort study reported that prenatal exposure to Mn-contaminated water was associated with increased conduct issues in boys and reduced prosocial behavior in girls by the age of 10 (Rahman et al., 2017). Other cross-sectional studies found significant correlations between higher Mn levels in tap water and impaired memory, attention deficits, and lower IQ scores (Ji et al., 2018; Tjalkens et al., 2017). More recent research suggests that the reduction in IQ scores is more pronounced in girls, indicating potential gender differences (Rahman et al., 2017; Dion et al., 2018; Bouchard et al., 2018). Cognitive function is not the only aspect impaired, as a cross-sectional study of toddlers in Bangladesh reported an inverse-U relationship between water Mn levels and fine motor scores (Rodrigues et al., 2016). Similarly, another study found an 11% reduction in motor scores after a ten-fold increase in Mn intake from tap water (Oulhote et al., 2014).

These findings highlight that early-life Mn exposure has significant neurodevelopmental implications, with the developing brain being especially sensitive to Mn accumulation. Human breast milk contains very low Mn concentrations (∼3–6 μg/L), whereas many infant formulas are fortified to hundreds or even thousands of µg/L (∼160–2,800 μg/L), providing approximately 28–520 times more Mn than breast milk in early infancy (Mitchell et al., 2021; Mitchell et al., 2020; Frisbie et al., 2019; Darr and Hamama, 2025). Because infants have immature biliary excretion and an incompletely developed blood–brain barrier, they are particularly susceptible to Mn overload. Epidemiological studies have associated elevated Mn exposure in childhood with reduced IQ and attentional performance (Kullar et al., 2019). Consequently, experts have cautioned that the markedly higher Mn content of formula—especially when prepared with Mn-rich water—may present an avoidable risk and warrants updated regulatory guidance (Mitchell et al., 2020; Sauberan and Katheria, 2025).

Beyond direct neurotoxicity, dietary Mn exposure during infancy may also influence intestinal ecology and developmental programming. Feeding mode shapes the early gut environment and microbiota composition (Lopez Leyva et al., 2025; Flores Ventura et al., 2024; Ma et al., 2020), which in turn may impact immune and neurodevelopmental outcomes later in life. Newborns naturally exhibit high intestinal calprotectin, which chelates Zn and Mn to create a metal-limited niche (Günaydın Şahin et al., 2020). In a recent study, formula-fed infants showed higher stool metal levels than breastfed infants despite similarly elevated calprotectin, accompanied by an enrichment of metal-tolerant genera (*Klebsiella*, *Enterobacter*, *Enterococcus*), whereas breastfed infants were enriched for Bifidobacterium and had fewer potentially pathogenic Gram-negative species (Ocaña et al., 2024). These findings suggest that differences in dietary Mn and other fortified metals, rather than inflammation, may shape early microbial composition and potentially influence immune and neurodevelopmental trajectories.

#### 5.1.2 Inhalation

Overexposure to Mn can also occur through environmental inhalation of Mn dust, particularly among workers in the mining, welding, and smelting industries. Mn levels in ambient air range between 0.009 µm^3^ to 0.085 µm^3^ in residential areas, mostly contributed by vehicle exhaust, whereas in industry, levels can go over 0.3 µm^3^, the minimum risk level for chronic Mn inhalations (Shaffer et al., 2023; EPA). A recent study revealed that ambient Mn concentration was associated with thinner grey matter thickness in men, suggesting cortical atrophy (Woo et al., 2023). A cross-sectional study involving 811 welders exposed to Mn fumes found a significantly higher prevalence of Parkinsonism compared to a reference group (Racette et al., 2012). Similarly, a survey of 505 welders reported a significantly increased incidence of tremors and muscle weakness compared to the general population (Zhang et al., 2017). Additionally, a recent meta-analysis concluded that occupational Mn overexposure is linked to lower cognitive performance, including deficits in memory, attention, and processing speed (Vlasak et al., 2023). Further supporting these findings, another study identified an inverse relationship between blood Mn levels and working memory in welders (Tsuji et al., 2023). A recent whole-brain MRI study in welders revealed that Mn accumulation expanded beyond the basal ganglia into the cerebellum and frontal cortex *via* white tracts, regions associated with motor and cognitive function (Monsivais et al., 2024). The latest study found that Mn brain accumulation varied with welding methods and correlated with Mn in red blood cells (Thunberg et al., 2024).

### 5.2 Mechanisms of Mn neurotoxicity

Mn toxicity affects neurotransmitters, neurons, and glial cells through mechanisms that include oxidative stress, inflammation, and apoptosis. Excess Mn can disrupt glutamate homeostasis by impairing synaptic reuptake, leading to glutamate excitotoxicity, neuronal overexcitation, and damage (Johnson et al., 2018; Erikson and Aschner, 2002; Hazell and Norenberg, 1997; Sidoryk-Węgrzynowicz et al., 2009; Nicos et al., 2024; Choi, 1988). This process has been implicated in many neurodegenerative disorders, including Parkinson’s and Alzheimer’s disease (Mehta et al., 2013). Astrocytes, which preferentially accumulate Mn due to the presence of high-affinity Mn transporters (Aschner et al., 1992; Sidoryk-Wegrzynowicz and Aschner, 2013), are primarily responsible for the glutamate uptake *via* GLAST and GLT (Rothstein et al., 1996; Mahmoud et al., 2019). *In vitro,* Mn overexposure reduces transporter expression and glutamate uptake (Erikson and Aschner, 2002; Hazell and Norenberg, 1997; Lee et al., 2009). Similarly, decreased EAAT1/EAAT2 (human homologs of GLAST/GLT1) mRNA and protein levels, as well as glutamate uptake, were observed in human astrocytes (Johnson et al., 2018). However, these *in vitro* studies often use relatively high Mn concentrations (up to 1 mM), exceeding physiological brain Mn levels, but acute culture systems generally require higher doses to reveal mechanistic effects in the absence of the physiological environment. Importantly, these findings were later corroborated by *in vivo* studies where both acute (single high-dose striatal injection) and sub-chronic (21 days of 30 mg/kg intranasal Mn) exposure reduced GLAST/GLT-1 expression and were associated with motor deficits, at doses approximating human Mn exposure (Johnson et al., 2018; Pajarillo et al., 2018). Recent studies have proposed possible mechanistic pathways contributing to the reduction in glutamate transporter expressions. Mn increases NFκB, which upregulates the transcriptional repressor YY1, suppressing GLAST/GLT-1 expression (Tinkov et al., 2021). Histone deacetylases (HDACs) can interact with YY1 to further inhibit expression (Karki et al., 2015; Karki et al., 2014). However, these mechanistic studies were derived from astrocyte cultures and remain to be validated *in vivo*. Mn overexposure also reduces glutamate receptor expression, NMDAR, in neurons *in vitro* and *in vivo*, potentially exacerbating glutamate-induced excitotoxicity (Wang et al., 2017; Xu et al., 2009). Together, these studies provide convincing evidence that astrocytes are vulnerable to Mn-induced disruption of glutamate uptake. Yet, much of the mechanistic findings rely on supraphysiological Mn doses *in vitro* and need confirmation at physiologically relevant Mn concentrations *in vivo*.

In addition to glutamate dysregulation, Mn overexposure causes neuroinflammation, largely mediated by astrocytes and microglia. Under normal conditions, glial cells become activated in response to ‘danger signals’ by releasing pro-inflammatory cytokines (TNF- α , ILs, and IFN- γ ), and nitric oxide as a defense mechanism, but excessive or prolonged activation becomes damaging (Tjalke et al., 2017). Glial activation is typically assessed by GFAP (astrocytes) and Iba1 (microglia). Mn overexposure through drinking water (Krishna et al., 2014), intraperitoneal injection (Cong et al., 2021; Tomás-Camardiel et al., 2002), and intragastric gavage (Moreno et al., 2009) increased GFAP and Iba1 expression, indicating glial activation. Notably, these routes are physiologically relevant, and Mn doses are physiologically relevant and also a good model of human Mn. Region-specific effects have also been reported, with astrocyte activation largely restricted to the striatum and substantia nigra, suggesting that Mn may target the nigrostriatal dopamine system (Tomás-Camardiel et al., 2002). Activated glia further release inflammatory cytokines and nitric oxide (Krishna et al., 2014; Cong et al., 2021; Sarkar et al., 2018; Kirkley et al., 2017; Popichak et al., 2018; Liu et al., 2006; Zhang et al., 2010). Early-life Mn overexposure increased glial activation and nitric oxide synthase expression in adult mice, highlighting developmental sensitivity to Mn overexposure (Moreno et al., 2009). Co-culture studies suggest microglia can amplify astrocyte activation after Mn overexposure, indicating communication between glial cells (Kirkley et al., 2017). NFκB has been identified as a key mediator of Mn-induced neuroinflammation (Popichak et al., 2018; Moreno et al., 2008; Kirkley et al., 2019; Hammond et al., 2020). *In vitro* pharmacological inhibition of NFκB in microglia blocked astrocyte inactivation, again, suggesting microglia-astrocyte interaction (Kirkley et al., 2017). Later, the same group discovered that mice that lacked NFκB in astrocytes had reduced Mn-induced glial activation and neuronal loss (Hammond et al., 2020). More recently, Mn was found to increase LRRK2 expression, a protein associated with Parkinson’s Disease, *in vitro* and *in vivo*, while LRRK2 deletion in microglia reduced cytokine release *in vitro* (Chen J. et al., 2018; Kim et al., 2019). Collectively, these studies provide strong evidence that Mn activates astrocytes and microglia, possibly through NFκB- or LRRK2-dependent pathways, that glia-glia interactions amplify responses, and that early-life exposure increases long-term susceptibility.

More recent efforts have examined the link between Mn overexposure, neuroinflammation, and exosomes. Specifically, Mn overexposure has been shown to promote the exosomal transmission of α-synuclein between neurons and microglia, triggering neuroinflammatory responses *in vitro* and *in vivo* (Harischandra et al., 2019; Sarkar et al., 2019). The latest study by the same group suggests that this effect may result from Mn impairing lysosomal protein degradation (Rokad et al., 2024). These results highlight the exosome-mediated pathway as a novel mechanism by which Mn impairs proteostasis and contributes to neurotoxicity.

Mn overexposure also affects the nigrostriatal dopaminergic system. Mn predominantly accumulates in the basal ganglia (Wei et al., 2021; Khan et al., 2020; Dietz et al., 2001; Josephs et al., 2005; Uchino et al., 2007), a region rich in dopamine and known to control motor function (Fazl and Fleisher, 2018). Early studies discovered that dietary Mn overexposure during the first 20 days of birth reduced striatal dopamine release with accompanying developmental deficits during adolescence (Tran et al., 2002). However, the Mn concentration given was very high (up to 500 µg per day). Later studies confirmed the impaired release of dopamine and its metabolites in the basal ganglia in rodents and non-human primates. Robison et al. demonstrated in rats that Mn accumulates in the dopaminergic cells of the substantia nigra pars compacta, at around 40–200 µM (Robison et al., 2015). They injected Mn intraperitoneally at a dose of 6 mg/kg which they measured blood Mn and which was at a similar level to human studies of Mn toxicity. Another study found that three sub-acute injections of high dose Mn (50 mg/kg) resulted in Mn still being elevated 21 days after treatment stopped and but dopamine release was only significantly lower after 7 days of treatment cessation, suggesting a delayed response (Khalid et al., 2011). However, the injection method is not physiologically relevant, and measurements were only done in male mice. Recent studies using intranasal injections of Mn at 30 days and 90 days are good models of chronic overexposure of Mn through inhalation. They showed Mn overexposure reduced dopamine concentrations in the striatum, accompanied by impaired motor performance (Ivleva et al., 2022; Ivleva et al., 2020). Another study demonstrated that oral exposure to high doses of Mn during the first 20 days of postnatal life reduces dopamine release later in young adolescent rats (Conley et al., 2020). Studies have also revealed Mn to decrease dopamine receptors (Conley et al., 2020; Song et al., 2016; Kern et al., 2010) and dopamine transporter expression (Roth J. A. et al., 2013; McDougall et al., 2008; Saputra et al., 2016).

In addition to dopamine release, the loss of dopaminergic neurons, a hallmark of Parkinson’s Disease, has also been examined in the context of Mn overexposure. One human study used PET imaging to show pre-synaptic dopaminergic dysfunction in Mn-exposed workers (Criswell et al., 2018). In addition, Mn overexposure may affect tyrosine hydroxylase (TH), an enzyme required for dopamine synthesis in dopaminergic terminals. Imaging techniques for TH are often used as a marker for dopaminergic neuron integrity. Mn was found to reduce TH expression in dopaminergic neurons *in vitro,* but at a high dose (Stanwood et al., 2009). In the same study, they also showed that after chronic Mn injection (daily for 30 days) in mice, there was a reduction in TH-positive cells in the substantia nigra. Similarly, the same dose of Mn was injected every 10 days for 150 days and also resulted in fewer TH-positive cells with impaired motor activity (Fan et al., 2020). Intranasal instillation at a higher level led to lower TH expression in nigrostriatal regions (Ivleva et al., 2022). Previous studies found that short-term neonatal exposure to Mn can lead to striatal TH reduction later in life in rats, though Mn doses were high (Conley et al., 2020; Peres et al., 2016). Mn was recently found to affect TH expression at a transcriptional level through the downregulation of the RE1-silencing transcription factor (REST) (Pajarillo et al., 2020b). Interestingly, 7 days of relatively low Mn exposure reduced TH-expressing cells in neuron-glial cultures, not in neuron cultures alone, indicating glial cells may exacerbate Mn toxicity in neurons (Zhang et al., 2010). However, the conclusions of the effect of Mn on TH expression remain divided. In macaques, chronic low-level Mn exposure reduced dopamine release and led to motor deficits but no change in striatal TH expression (Guilarte et al., 2006b; Guilarte et al., 2008). Based on these findings, the authors concluded that dopaminergic neuron integrity was preserved, unlike in Parkinson’s Disease. Another study found that intranasal delivery of Mn led to motor deficits with TH loss in the olfactory bulb but not in the striatum (Sarkar et al., 2018). A lack of TH expression change was also found in *Zip14* KO mice that displayed high brain Mn levels (Rodichkin et al., 2021). While there is an inconsistency in the findings concerning TH expression, it is clear that Mn overexposure has a detrimental effect on dopamine activity in the nigrostriatal region. A recent study also discovered that Mn accumulated in dopamine neurons and increased excitability, reduced amplitude, and half-width of action potentials (Lin et al., 2020). These findings provide another mechanism for Mn-induced dysfunction of dopamine neurons rather than the direct loss of these neurons.

Furthermore, Mn preferentially accumulates in the mitochondria and causes mitochondrial dysfunction (Tinkov et al., 2021; Lai et al., 1999; Gunter et al., 2009). In a cell model of mitochondrial dysfunction, Mn-induced toxicity was exacerbated, as shown by worsened motor performance, more inflammation, loss of nigral TH neurons, and lower dopamine levels (Langley et al., 2018). Studies found loss of mitochondrial membrane potential (Huang et al., 2021; Gugnani et al., 2018; Bonke et al., 2016; Rama Rao and Norenberg, 2004; Malecki, 2001; Maddirala et al., 2015; Yin et al., 2008; Milatovic et al., 2007) and lowered activity in electron transport chain complex I and II (Zhang et al., 2003), in neurons and glia. However, most of these findings are derived from *in vitro* or *ex vivo* studies, so studies need to be *in vivo* to fully capture mitochondrial changes during chronic Mn overexposure at a physiological level. With mitochondrial dysfunction, Mn can induce oxidative stress as shown by the increase in ROS *in vivo* (Cong et al., 2021; Zhang et al., 2003) and *in vitro* (Huang et al., 2021; Bonke et al., 2016; Maddirala et al., 2015; Milatovic et al., 2007; Neely et al., 2017). Huang et al. discovered that MnCl_2_ treatment to SH-SY5Y, a common cell model for dopaminergic neurons, resulted in mitochondrial membrane potential loss, ROS production, and apoptosis (Lee et al., 2009). They found that the deletion of a mitophagy protein, BNIP3, can reverse Mn-induced ROS generation. The increased oxidative stress can also be attributed to the reduced antioxidant levels. Cong et al. showed that Mn injections for 6 weeks reduced superoxide dismutase, catalase, and glutathione (GSH) activity (Cong et al., 2021). The same observations were found in rat brains *in vivo* (Morello et al., 2007) and in dopamine neurons (Neely et al., 2017) and SH-SY5Y cells *in vitro* (Maddirala et al., 2015). Collectively, the evidence supports mitochondrial dysfunction and oxidative stress as markers of Mn neurotoxicity. Yet, important gaps remain regarding the mechanism by which Mn causes these.

Furthermore, Mn can induce apoptosis. *In vitro* studies first showed that Mn exposure (above 10 µM) induces apoptosis in dopaminergic neurons (Latchoumycandane et al., 2005) and the PC12 cell line, a common model for neurons (Hirata, 2002; Kitazawa et al., 2005). Latchoumycandane et al. found that apoptosis is caused by the release of cytochrome c from the mitochondria, leading to the cleavage of caspase 3 and PKC δ, and finally DNA fragmentation (Latchoumycandane et al., 2005). Similarly, Kitazawa et al. concluded that Mn-induced apoptosis involved PKC δ and Bcl2, a regulator of apoptosis (Kitazawa et al., 2005). Mn-induced apoptosis was confirmed by later studies *in vitro* (Tinkov et al., 2021; Harischandra et al., 2015; Yagyu et al., 2020; Li et al., 2010; Liu et al., 2020). Yagyu et al. showed that 500 µM MnCl_2_ exposure increased caspase 3 cleavage and reduced cell viability in PC12 cells (Yagyu et al., 2020). Interestingly, these effects were reversed by a protein kinase R inhibitor, indicating the involvement of protein kinase R in Mn-induced apoptosis. Mn-induced apoptosis is also seen *in vivo* after intrastriatal injections (Alaimo et al., 2014) and intraperitoneal injections (Liu et al., 2020). However, the intrastriatal approach is an acute, high-dose exposure that bypasses normal physiological regulation and therefore does not accurately model environmental overexposure. Recent studies discovered other pathways for Mn-induced apoptosis. Mn can reduce mitochondrial fusion proteins *in vitro* and *in vivo*, which can lead to neuronal apoptosis (Alaimo et al., 2014; Liu X. et al., 2017). Mn-induced ER stress may also be a catalyst for apoptosis. Mn overexposure led to increased expression of proteins in the ER stress pathway, while ER stress inhibitor reduced Mn-induced apoptosis (Liu et al., 2020). Moreover, Mn also downregulates the expression of Bcl2, an anti-apoptotic protein, in this case, consequently resulting in more apoptosis (Yang Y. et al., 2019). Notably, recent studies found that Mn overexposure can also increase cellular Fe uptake, reduce antioxidant levels, resulting in increased lipid peroxidation and ferroptosis in both cells and mouse models (Chen et al., 2024; Wang et al., 2024). Overall, these results suggest that Mn overexposure triggers multiple programmed cell death pathways, including apoptosis and ferroptosis, through mitochondrial dysfunction, ER stress, and impaired redox balance.

### 5.3 Mn toxicity in other systems

Although the effects of Mn overexposure on other bodily systems are less explored, both human and animal studies suggest that excess Mn may impact the cardiovascular system and reproductive systems. Case studies of Mn alloy works have reported lower blood pressure, altered heart rate, and changes in ECG patterns (Jiang et al., 2005). These findings are supported by animal studies, where Mn overexposure reduced heart rate, decreased cardiac contractility, and also led to ECG abnormalities (Jiang et al., 2005). In addition to the cardiovascular system, Mn overexposure has also been linked to reproductive health effects. A study of male workers overexposed to Mn found lower testosterone levels and reduced sperm motility (Yang H. et al., 2019). Similarly, an animal study on cocks demonstrated that Mn supplementation led to a decline in reproductive hormone levels (Liu et al., 2013). In female rats, early-life Mn overexposure disrupted hormonal production and led to abnormal reproductive tissue development (Kim et al., 2012). Mn toxicity has also been investigated in the lungs, liver, and kidneys. Animal and *in vitro* studies have reported a wide range of Mn-induced pulmonary effects, including lung damage, inflammation, alveolar macrophage infiltration, and oxidative stress (Gandhi et al., 2022). Similarly, Mn overexposure has been associated with liver damage, impaired liver function, histopathological changes, oxidative stress, and mitochondrial dysfunction (Gandhi et al., 2022). Kidney-related toxicity has also been observed, with Mn overexposure leading to renal inflammation, impaired kidney function, and tissue damage (Gandhi et al., 2022).

Additionally, excessive Mn has been linked to metabolic disorders, including type II diabetes, obesity, and cardiovascular disease, with oxidative stress proposed as a key contributing factor (Li and Yang, 2018). However, epidemiological studies on this topic have yielded conflicting results. While some studies found a positive association between higher Mn intake and an increased risk of metabolic syndromes, others reported the opposite effect, with some studies even suggesting gender-specific differences (Li and Yang, 2018). These discrepancies may stem from confounding factors, making it challenging to draw definitive conclusions.

## 6 Disease modulation of Mn transport and metabolism

Mn transport and metabolism are dynamically regulated across diverse disease states.

In the gastrointestinal tract, chronic inflammation alters Mn absorption and epithelial barrier function. A *SLC39A8* (ZIP8 A391T) variant identified in genome-wide studies disrupts the regulation of multiple metals, including Mn, and increases susceptibility to Crohn’s disease (Li et al., 2016). Similarly, intestinal *Zip8* KO mice exhibit systemic Mn deficiency and aggravated colitis, which can be rescued by inhibition of the downstream sphingolipid enzyme ACER1 (Choi et al., 2024b). In human IBD, ZIP8 expression is induced by interferon-γ and correlates with inflammatory markers such as tumor necrosis factor-α and calprotectin (Melia et al., 2019). Moreover, Mn-deficient diets exacerbate colitis in mice, whereas Mn supplementation restores epithelial integrity and reduces mucosal injury (Choi et al., 2020). These findings demonstrate that intestinal inflammation modulates metal transporter expression and Mn distribution, influencing both local and systemic homeostasis.

Mn metabolism is also shaped by immune-mediated metal redistribution through nutritional immunity. During infection or inflammation, host defense mechanisms restrict metal availability to limit microbial growth. The neutrophil protein calprotectin chelates Mn and Zn in the gut lumen and extracellular spaces, while the macrophage transporter Nramp1 (SLC11A1) exports Mn and Fe from phagosomes, depriving pathogens of essential cofactors (Diaz-Ochoa et al., 2016; Cellier et al., 2007). Together, these mechanisms create localized metal deprivation that suppresses microbial proliferation and modulates Mn availability within host tissues.

In metabolic syndrome and non-alcoholic fatty liver disease (NAFLD), Mn homeostasis is also altered. Elevated serum Mn is positively associated with NAFLD prevalence (Xue and Chen, 2025; He et al., 2025). Experimental studies suggest that Mn excess promotes hepatic steatosis through oxidative stress and insulin resistance, while Mn deficiency impairs mitochondrial antioxidant defense and lipid metabolism (Hu et al., 2025; Liu et al., 2025). This U-shaped relationship indicates that both Mn deficiency and overload can compromise metabolic function.

Together, these findings illustrate that Mn homeostasis is not static but is dynamically reshaped by disease, reflecting adaptive and maladaptive responses across organ systems.

## 7 Discussion

Mn is an essential micronutrient and trace element required for mitochondrial function, antioxidant defense, and cellular metabolism. However, the balance between Mn sufficiency and toxicity is narrow, and dysregulation contributes to a spectrum of pathologies ranging from neurodegeneration to metabolic and inflammatory diseases. This review highlights the integrated roles of Mn transporters, including ZIP8, ZIP14, and ZNT10, in maintaining systemic and cellular Mn homeostasis and how their dysfunction leads to tissue-specific vulnerability.

Despite substantial progress, several important questions remain. First, the spatial organization and regulation of Mn transporters across polarized tissues such as the intestine, liver, and brain are incompletely defined. Clarifying the apicobasal localization of ZIP8, ZIP14, and ZNT10 and understanding their coordinated regulation under Mn deficiency or overload will be critical to delineate how Mn fluxes are maintained during metabolic or inflammatory stress. Moreover, disease-induced alterations in Mn transporters and metabolism, and how these changes, in turn, exacerbate disease progression, remain largely unexplored. Understanding these reciprocal interactions will be key to determining whether Mn dysregulation is a driver or consequence of pathology.

Second, Mn metabolism cannot be studied in isolation because of its shared transport and signaling pathways with other transition metals. The molecular basis of Mn–Zn–Fe crosstalk remains unclear. Defining how fluctuations in one metal influence the homeostasis and signaling of others will refine our understanding of metal–metal interactions in disease and may inform strategies such as metal supplementation or chelation therapy.

Finally, Mn imbalance extends beyond the nervous system to metabolic, reproductive, and cardiovascular dysfunctions. The mechanisms integrating Mn with hormonal regulation, oxidative stress, and energy metabolism remain to be fully elucidated. Multi-organ and sex-specific studies examining intestinal, hepatic, and neural Mn regulation will be essential to construct a systems-level model of Mn metabolism.

Taken together, these insights emphasize the need for a systems approach to fully elucidate Mn biology. Integrating molecular genetics, spatial imaging, and multi-omics, including transcriptomics, proteomics, metabolomics, and metallomics, will enable comprehensive mapping of Mn transport and utilization across tissues and disease states.
